# Качество жизни пациентов с первичным гиперпаратиреозом после хирургического лечения

**DOI:** 10.14341/probl12825

**Published:** 2022-01-11

**Authors:** Т. П Никитина, И. Н. Гладкова, В. Ф. Русаков, Р. А. Черников, Ю. В. Карелина, С. М. Ефремов, Т. И. Ионова

**Affiliations:** Санкт-Петербургский государственный университет, Клиника высоких медицинских технологий им. Н. И. Пирогова; Санкт-Петербургский государственный университет, Клиника высоких медицинских технологий им. Н. И. Пирогова; Санкт-Петербургский государственный университет, Клиника высоких медицинских технологий им. Н. И. Пирогова; Санкт-Петербургский государственный университет, Клиника высоких медицинских технологий им. Н. И. Пирогова; Санкт-Петербургский государственный университет, Клиника высоких медицинских технологий им. Н. И. Пирогова; Санкт-Петербургский государственный университет, Клиника высоких медицинских технологий им. Н. И. Пирогова; Санкт-Петербургский государственный университет, Клиника высоких медицинских технологий им. Н. И. Пирогова

**Keywords:** первичный гиперпаратиреоз, паратиреоидэктомия, качество жизни и симптомы, опросник оценки качества жизни

## Abstract

**ОБОСНОВАНИЕ:**

ОБОСНОВАНИЕ: Для комплексной оценки эффекта хирургического лечения у больных с первичным гиперпаратиреозом (ПГПТ), а также для мониторинга состояния больных после операции представляется актуальным проведение оценки качества жизни и симптомов у пациентов до и после операции.

**ЦЕЛЬ:**

ЦЕЛЬ. Цель данного исследования заключалась в изучении особенностей изменения качества жизни и симптомов у больных с разными формами ПГПТ после проведения хирургического лечения.

**МАТЕРИАЛЫ И МЕТОДЫ:**

МАТЕРИАЛЫ И МЕТОДЫ. В рамках проспективного наблюдательного исследования пациенты заполняли общий и специальный опросники для оценки качества жизни и оценивали наличие и выраженность симптомов ПГПТ до операции, через 3 и 12 мес после паратиреоидэктомии (ПТЭ). Статистический анализ проводили с помощью t-критерия Стьюдента или непараметрического критерия Вилкоксона, метода обобщенных оценочных уравнений (generalized estimating equations, GEE), корреляционного анализа, критерия χ2 и критерия Мак-Немара.

**РЕЗУЛЬТАТЫ:**

РЕЗУЛЬТАТЫ. В исследование включены 72 пациента (средний возраст 52 года, 97,2% – женщины) с манифестной (68,1%) и бессимптомной (31,9%) формами заболевания. До проведения ПТЭ у пациентов с ПГПТ показано выраженное нарушение ролевого функционирования, физического и социального функционирования, а также жизнеспособности. У половины пациентов выявлены такие существенно выраженные симптомы, как слабость, усталость, когнитивные нарушения, перемены в настроении, а также суставные и костные боли; продемонстрирована связь между испытываемыми симптомами и степенью нарушения качества жизни до операции. Через 3 мес после ПТЭ у больных ПГПТ показано улучшение как по физическому, так и психологическому компонентам качества жизни. Положительные изменения качества жизни установлены как при манифестной, так и бессимптомной форме заболевания; они сохранялись в течение 12 мес после операции. Также в течение 12 мес после ПТЭ отмечено существенное уменьшение таких актуальных для ПГПТ симптомов, как слабость, усталость, забывчивость и перемены в настроении. Успешное хирургическое лечение с нормализацией уровня паратиреоидного обмена способствует улучшению качества жизни больных ПГПТ, независимо от формы заболевания.

**ЗАКЛЮЧЕНИЕ:**

ЗАКЛЮЧЕНИЕ. Полученные результаты демонстрируют положительный эффект хирургического лечения с точки зрения пациента и подтверждают целесообразность оценки качества жизни как на этапе принятия решения при выборе хирургической тактики, так и в составе комплексной оценки эффективности терапии при определении степени восстановления разных аспектов функционирования у пациентов после операции.

## Abstract (en)

**BACKGROUND:**

BACKGROUND: For a comprehensive assessment of the effect of surgery in patients with primary hyperparathyroidism (PHPT), as well as for monitoring the condition of patients after treatment, it sounds reasonable to evaluate quality of life (QoL) and symptoms in PHPT patients before and after surgery.

**AIM:**

AIM: The aim of this study was to assess changes in the QoL and symptoms in patients with PHPT after surgery.

**MATERIALS AND METHODS:**

MATERIALS AND METHODS: During prospective observational study, patients filled out QoL questionnaires and evaluated the presence and severity of their symptoms prior to parathyroidectomy (PTE) and 3, 12 months after surgery. Statistical analysis included the following methods: Student’s t-test or Wilcoxon’s non-parametric test, the generalized estimating equations (GEE), correlation analysis, χ2 and McNemar tests.

**RESULTS:**

RESULTS: The study included 72 patients (mean age 52 years, 97.2% female) with symptomatic (68.1%) and asymptomatic (31.9%) PHPT. Before surgery patients with PHPT exhibited significantly decreased role functioning, physical and social well-being, and vitality. Half of PHPT patients experienced moderate-to-severe symptoms such as weakness, fatigue, loss of concentration, mood changes, as well as joint and bone pain; the association between symptoms experienced and the extent of QoL impairment before surgery was shown. Three months after PTE improvement in both physical and psychological components of QoL was shown. Positive QoL changes were demonstrated in patients with both symptomatic and asymptomatic PHPT and they preserved for 12 months after surgery. Also within 12 months after PTE significant decrease in PHPT-associated symptoms such as weakness, fatigue, loss of concentration and mood changes was found.

**CONCLUSION:**

CONCLUSION: The results obtained demonstrate efficacy of PTE from the patient’s perspective and confirm the value of QoL assessment in PHPT patients in management of this patients’ population both for decision making and for evaluation of benefits of surgery and the degree of recovery of patients at long term follow-up.

## ОБОСНОВАНИЕ

Хирургическое лечение в настоящее время является основным методом лечения первичного гиперпаратиреоза (ПГПТ). Паратиреоидэктомия (ПТЭ) показана всем пациентам с симптомной (манифестной) формой заболевания и при малосимптомной (синоним: мягкой) форме пациентам моложе 50 лет, а также при высоком уровне кальция крови с угрозой развития гиперкальциемического криза и выраженной гиперкальциурией [1][2]. Кроме того, хирургическое лечение может быть рекомендовано в случае бессимптомного ПГПТ и отсутствия показаний к ПТЭ при желании самого пациента, при этом необходима оценка соотношения риска/пользы от операции [1][2]. Преимущества оперативного лечения заключаются в нормализации уровня кальция и устранении ассоциированных с гиперкальциемией симптомов, значимом улучшении состояния костной ткани и почек, а также улучшении со стороны сердечно-сосудистой системы и когнитивных функций у абсолютного большинства прооперированных пациентов [1]. Принимая во внимание значительное число пациентов с бессимптомным течением ПГПТ и отсутствием типичных клинических проявлений заболевания, но при этом с наличием нейрокогнитивных симптомов, остается актуальным вопрос о целесообразности хирургического вмешательства в таких случаях [3]. В ряде исследований подтверждено уменьшение нейрокогнитивных симптомов после ПТЭ, в связи с чем нейропсихологическое тестирование может быть рекомендовано в качестве одного из компонентов обследования пациентов с ПГПТ для определения показаний к хирургическому лечению с целью предотвращения дальнейшего ухудшения когнитивных функций [4].

Опубликованные данные демонстрируют положительную динамику показателей качества жизни у больных ПГПТ в разные сроки после хирургического лечения [5–13]. Для оценки качества жизни в данных работах авторы использовали как общие опросники качества жизни (SF-36, EQ-5D, 15-D), так и специальные опросники для больных ПГПТ (PHPQoL, PAS). Перечень наиболее актуальных задач, решаемых в ходе данных исследований, включает, помимо анализа степени восстановления разных аспектов функционирования после операции, изучение закономерностей изменения качества жизни в разные сроки после хирургического лечения, в том числе у пациентов с малосимптомным течением болезни и с манифестной формой ПГПТ, а также с разной степенью гиперкальциемии [11][13][14]. Отдельные исследования проведены по определению предикторов улучшения качества жизни у больных после операции [10–12]. Обращают внимание результаты рандомизированных исследований, свидетельствующие об улучшении качества жизни пациентов с бессимптомным ПГПТ после хирургического лечения [1][5][6]. Среди отечественных работ данных по изучению качества жизни после ПТЭ у больных ПГПТ в настоящее время крайне мало. В недавно опубликованной работе отечественных авторов, направленной на выбор оптимального хирургического доступа при выполнении ПТЭ у больных ПГПТ, сопровождающегося выраженной положительной динамикой качества жизни у больных после операции, авторы подчеркивают важное практическое значение необходимости использования специальных инструментов оценки качества жизни при ведении больных ПГПТ до и после операции для совершенствования отечественной системы медицинской помощи данной категории пациентов [15]. Особо отметим, что использование информации, полученной напрямую от пациента, при анализе клинических проявлений заболевания и оценке динамики симптомов в процессе лечения способствует реализации пациент-ориентированного подхода при ведении больных ПГПТ [16].

Для оценки изменений различных аспектов функционирования пациентов после медицинских вмешательств информативным является совместное использование общего и специального опросников качества жизни в дополнение с оценкой актуальных симптомов [16]. В настоящее время при ПГПТ предложены к применению специальный опросник оценки качества жизни при ПГПТ — PHPQoL [17] и специальный опросник оценки симптомов при ПГПТ — PAS [18]. Оба инструмента апробированы в популяции больных ПГПТ и рекомендованы их авторами к использованию в научных исследованиях и рутинной клинической практике [14][17][18]. Разработка и апробация русскоязычных версий данных опросников в группе больных ПГПТ проведены в Клинике Высоких медицинских технологий им. Н.И. Пирогова СПбГУ [19]. Таким образом, оценка качества жизни и нейропсихологических симптомов до и после хирургического лечения при ПГПТ могут быть рекомендованы для комплексной оценки эффекта терапии, а также мониторинга состояния больного после операции, в том числе в реальной клинической практике.

## ЦЕЛЬ ИССЛЕДОВАНИЯ

Целью настоящего исследования являлось изучение особенностей изменения качества жизни и симптомов у пациентов с разными формами ПГПТ до и после хирургического лечения.

## МАТЕРИАЛЫ И МЕТОДЫ

Место и время проведения исследования

Место и время проведения. Исследование проводили в период с сентября 2019 г. по февраль 2021 г. на базе отделения эндокринной хирургии Клиники высоких медицинских технологий им. Н.И. Пирогова СПбГУ, Санкт-Петербург.

Методы

В одноцентровое наблюдательное проспективное исследование включали пациентов 18 лет и старше с подтвержденным диагнозом ПГПТ, при наличии показаний для проведения хирургического лечения, а также при условии согласия на участие в исследовании и способности пациентов заполнить опросники. Пациенты проходили лечение в отделении эндокринологии и эндокринной хирургии Клиники высоких медицинских технологий им. Н.И. Пирогова СПбГУ. Перед началом исследования было получено письменное информированное согласие на участие в исследовании от каждого пациента. К критериям исключения относили наличие сопутствующих заболеваний, симптомы которых, по мнению врача, преобладали над симптомами основного заболевания, а также наличие у пациентов выраженных ментальных нарушений, препятствующих заполнению опросников. Обязательным критерием включения являлось достижение нормокальциемии и нормализация уровня паратгормона в послеоперационный период.

Все пациенты были прооперированы в период с сентября 2019 г. по февраль 2021 г. Клинико-лабораторное обследование у всех пациентов до операции включало определение уровня ионизированного кальция, фосфора и паратгормона (ПТГ) сыворотки крови, скорости клубочковой фильтрации, клиренса креатинина, суточной экскреции кальция и фосфора с мочой, ЭКГ, клинический анализ крови и общий анализ мочи и DEXA- или компьютерную остеоденситометрию. Также исследовался уровень ионизированного кальция, паратгормона через 1–4 ч после операции и затем ежедневно уровень кальция и креатинина, а также паратгормона — по показаниям. Всем пациентам проводилось гистологическое исследование операционного материала с целью морфологической верификации диагноза. При выписке из стационара после проведенной ПТЭ пациентам были даны рекомендации по приему препаратов кальция и активных форм витамина D под контролем показателей фосфорно-кальциевого обмена.

Для оценки качества жизни использовали общий опросник RAND SF-36 и специальный опросник для больных ПГПТ — PHPQoL, для оценки встречаемости и выраженности симптомов у больных ПГПТ — отдельные вопросы из специального опросника симптомов PAS. Пациенты заполняли опросники трижды — при поступлении в отделение эндокринологии и эндокринной хирургии до операции, через 3 и 12 мес после операции. На динамических точках пациенты заполняли опросники дистанционно в форме электронных анкет на основе интернет-ресурса.

PHPQoL (Primary Hypoparathyroidism Quality of Life questionnaire) — специальный опросник оценки качества жизни при ПГПТ [14, 17]. Он содержит 16 вопросов, первые 9 вопросов касаются физического функционирования больного, остальные 7 — эмоционального функционирования. Образец русской версии опросника представлен в Приложении 1. Варианты ответов представляют собой баллы по шкале Ликерта для оценки того, как часто беспокоила пациента та или иная проблема в течение последнего месяца (0 баллов — проблема была всегда, 1 балл — очень часто, 2 — время от времени, 3 — очень редко, 4 балла — проблемы никогда не было). Сумму баллов по шкалам Ликерта для 16 вопросов преобразуют с помощью процедуры стандартизации в суммарный показатель качества жизни, значения которого могут варьировать от 0 до 100 — чем выше суммарный показатель, тем лучше качество жизни. Формула для расчета суммарного показателя по опроснику PHPQoL представлена ниже.

СП = номинальное количество баллов по 16 вопросам/64 × 100.

Таким же способом рассчитывается физический компонент (ФК) качества жизни (стандартизированная сумма баллов по 9 вопросам опросника) и психический компонент (ПК) качества жизни (стандартизированная сумма баллов по 7 вопросам опросника) [14][17].

ФК = номинальное количество баллов по 9 вопросам/36 × 100.

ПК = номинальное количество баллов по 7 вопросам/28 × 100.

На основании суммарного показателя по опроснику PHPQoL можно определить клинически значимое улучшение качества жизни больных в процессе лечения, которое соответствует увеличению суммарного показателя на 9 и более баллов по сравнению с его исходным значением [14].

RAND SF-36 — широко распространенный общий опросник оценки качества жизни, используемый у здоровых людей и у пациентов с хроническими заболеваниями [20]. Инструмент предназначен для респондентов от 14 лет и состоит из 36 вопросов, которые формируют восемь шкал: физическое функционирование (ФФ), ролевое физическое функционирование (РФФ), боль (Б), общее здоровье (ОЗ), жизнеспособность (Ж), социальное функционирование (СФ), ролевое эмоциональное функционирование (РЭФ), психическое здоровье (ПЗ). Число вопросов в каждой из шкал опросника варьирует от 2 до 10, на каждый вопрос предлагаются различные варианты ответов. После проведения шкалирования (перевода необработанных данных в баллы качества жизни) результаты исследования выражают в баллах от 0 до 100 по каждой из 8 шкал. Чем выше балл по шкале опросника SF-36, тем лучше показатель качества жизни.

Опросник PAS — опросник для скрининга и мониторинга наиболее часто встречающихся у больных ПГПТ симптомов для применения в клинической практике [18]. Он включает 13 пунктов для оценки наиболее распространенных симптомов при гиперпаратиреозе: усталость, жажда, перемены в настроении, боль в суставах, постоянная раздражительность, плохое настроение/депрессия, слабость, кожный зуд, забывчивость, головная боль, боль в животе, боль в костях, проблемы при вставании из положения сидя. Симптомы количественно оцениваются по шкале от 0 до 100, «0» обозначает полное отсутствие симптома, «100» — максимальную выраженность симптома, которую можно себе представить. Значение в диапазоне от 40 до 100 баллов рассматривают как значительно выраженный симптом. В исследовании оценивали все 13 симптомов опросника PAS согласно их выраженности от 0 до 100 баллов. Расчет единого показателя выраженности симптомов, предусмотренный в опроснике PAS, не проводили. Для сравнения качества жизни пациентов до операции с условно здоровыми лицами была сформирована группа респондентов из базы популяционного исследования качества жизни на основании опросника RAND SF-36, которая была сопоставима с группой пациентов по полу и возрасту (n=72).

Статистический анализ. Данные представлены в виде количества наблюдений, среднего арифметического значения, стандартного отклонения, диапазона, квартилей и процентных долей. При выборе критерия проверки статистической значимости различий между анализируемыми показателями основывались на характере распределения данных, оцененном с помощью теста Колмогорова-Смирнова. При сравнении 2 групп пользовались критерием сравнения для двух несвязанных выборок — t-критерием Стьюдента или непараметрическим критерием Вилкоксона. При изучении показателей в динамике использовали метод «обобщенные оценочные уравнения» (generalized estimating equations, GEE). В группах больных с разными формами ПГПТ также оценивали клинически значимые изменения показателей качества жизни в динамике на основании расчета величины эффекта для повторных измерений (effect size for repeated measures, ES). Выделяли следующие градации величины эффекта: малый — 0,2≤ES<0,5; средний — 0,5≤ES<0,8; большой эффект — ES≥0,8. Для оценки связи между показателями использовали ранговую корреляцию Спирмена. Корреляционную связь рассматривали как очень слабую при r<0,3, слабую — при r=0,3–0,5, среднюю — при r=0,5–0,7, высокую — при r=0,7–0,9, очень высокую — при r>0,9. Сравнение пропорций в разных группах больных проводили с помощью критерия χ2, в динамике у одних и тех же пациентов — с помощью критерия Мак-Немара.

Все тесты двусторонние, различия между сравниваемыми группами признавали статистически значимыми при уровне p<0,05. Статистический анализ проведен с использованием программного обеспечения SPSS 23.0 и MedCalc.

Этическая экспертиза

Исследование одобрено Комитетом по биомедицинской этике Клиники высоких медицинских технологий им. Н.И. Пирогова СПбГУ (выписка из протокола №08/19 от 15.08.2019).

## РЕЗУЛЬТАТЫ

В исследовании приняли участие 72 пациента с ПГПТ, средний возраст больных (±стандартное отклонение среднего значения) — 51,8±10,4 года, 97,2% составили женщины. В табл. 1 представлена общая характеристика выборки пациентов.

**Table:** Таблица 1. Характеристика выборки

Показатели	Значение
Общее количество больных, n (%)	72 (100%)
Возраст, среднее значение (стандартное отклонение), лет	51,8 (10,4)
Женщины, n (%)Мужчины, n (%)	70 (97,2)2 (2,8)
ECOG*, n (%)0	23 (31,9)
1	48 (66,7)
2	1 (1,4)
Форма заболевания, n (%)Малосимптомная	23 (31,9)
Манифестная	49 (68,1)
Гиперкальциемия, n (%)Легкая (Са ионизированный до 1,49 ммоль/л)	47 (65,3)
Умеренная (Са ионизированный 1,5–1,79 ммоль/л)	22 (30,5)
Тяжелая (Са ионизированный выше 1,8 ммоль/л)	3 (4,2)
Артериальная гипертензия, n (%)	40 (55,6)
Хроническая сердечная недостаточность, n (%)	14 (19,4)
Сопутствующие заболевания**, n (%)	49 (68,1)

Длительность заболевания (от постановки диагноза до начала исследования) составила в среднем 12±16 мес (медиана — 7 мес). У более половины пациентов имелась манифестная форма ПГПТ (68,1%). Умеренная или тяжелая гиперкальциемия выявлена у 34,7% больных. До операции среднее значение ионизированного кальция составило 1,5±0,15 ммоль/л (норма 1,1–1,31), среднее значение ПТГ — 102,1±124,9 пг/мл (норма до 65 пг/мл). Размеры аденомы околощитовидной железы/желез находились в диапазоне 1–6 см. Остеопороз позвоночника/шейки бедра/лучевой кости выявлен у 22 пациентов (30,6%), мочекаменная болезнь — у 30 пациентов (41,7%), при этом хроническая почечная недостаточность (ХБП) ст. 2–4 — у 9 пациентов (12,5%), хроническая сердечная недостаточность — у 14 пациентов (19,4%).

Рассмотрим показатели качества жизни и спектр симптомов у пациентов до операции. В соответствии с данными специального опросника PHPQoL до операции суммарный показатель по опроснику составил 53,7±15,3 балла (диапазон 21,9–95,3 балла). При этом показатель физического компонента качества жизни — 54,5±17,5 балла; психического компонента качества жизни — 52,1±15,6 балла. Распределение пациентов согласно величине суммарного показателя качества жизни по опроснику PHPQoL до операции было следующим: 0–25 баллов — 2 пациента, 26–50 баллов — 29 пациентов, 51–75 баллов — 33 пациента, 76–100 баллов — 8 пациентов. Таким образом, у подавляющего количества пациентов показатели качества жизни по опроснику PHPQoL находились в среднем диапазоне (83%); у 40,3% больных отмечалось низкое качество жизни (26–50 баллов), а у 2,8% больных — очень низкое качество жизни (0–25 баллов) согласно специальному опроснику PHPQoL.

Также провели оценку качества жизни больных до операции с помощью общего опросника SF-36 и для определения степени нарушения разных аспектов качества жизни у пациентов сравнили показатели по шкалам данного опросника у больных ПГПТ с условно здоровыми респондентами того же пола и возраста, без ПГПТ и с нормальным фосфорно-кальциевым обменом. На рисунке 1 представлены показатели качества жизни у больных ПГПТ по опроснику SF-36 в сравнении с условно здоровыми респондентами.

Как видно на рис. 1, показатели качества жизни у больных ПГПТ до операции значимо ниже, чем у условно здоровых респондентов, в большей степени за счет ролевого (физического и эмоционального) функционирования, физического функционирования, социального функционирования и жизнеспособности (p<0,05). Таким образом, у больных ПГПТ до операции установлено существенное нарушение как физической, так и психической составляющих качества жизни.

**Figure:**
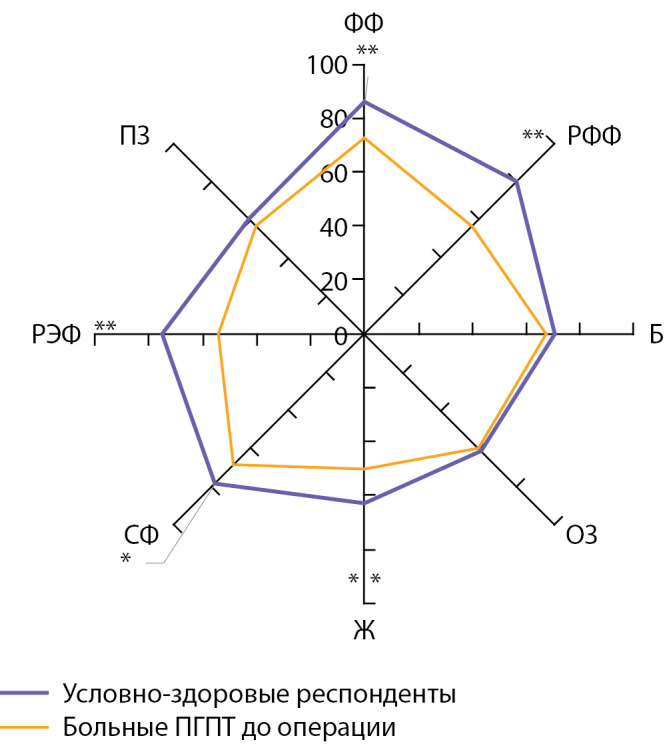
Рисунок 1. Показатели качества жизни у больных первичным гиперпаратиреозом до операции и условно здоровых респондентов по опроснику SF-36; *p<0,001; **p<0,05.Здесь и далее для рис. 2 и табл. 5-6: Шкалы SF-36: ФФ — физическое функционирование, РФФ — ролевое физическое функционирование, Б — боль, ОЗ — общее здоровье, Ж — жизнеспособность, СФ — социальное функционирование, РЭФ — ролевое эмоциональное функционирование, ПЗ — психическое здоровье.

При анализе профиля симптомов определено, что у больных ПГПТ до операции чаще всего регистрировались следующие симптомы — усталость (97,2% больных), забывчивость (95,8%), слабость (94,4%), перемены настроения (90%), плохое настроение/депрессия (87,3%), боли в суставах или/и в костях (84,7%), постоянная раздражительность (79,2%), головная боль (77,5%), жажда (69,4%) и проблемы при вставании из положения сидя (63,9%). Меньше половины больных испытывали боли в животе (42,3%) и кожный зуд (34,3%). Отдельно отметим, что у половины пациентов наблюдались значительно выраженные усталость, слабость, боли в суставах или/и в костях, забывчивость, а также перемены настроения (табл. 2). Также показано, что чем больше симптомов и проблем, связанных с заболеванием, испытывает пациент, тем хуже его показатели качества жизни по опроснику PHPQoL (r=-0,46; p<0,001). Таким образом, до операции актуальными симптомами у больных ПГПТ были слабость, усталость, боли в суставах или/и в костях, забывчивость и перемены настроения.

**Table:** Таблица 2. Характеристика симптомов у больных первичным гиперпаратиреозом до операции

Симптомы	Медиана,баллы	Нижний квартиль, баллы	Верхний квартиль, баллы
Усталость	60	45	80
Жажда	25	0	55
Перемены настроения	40	20	70
Боли в суставах	50	20	90
Постоянная раздражительность	35	10	60
Плохое настроение/депрессия	30	10	60
Слабость	50	30	80
Кожный зуд	0	0	20
Забывчивость	50	20	80
Головная боль	30	10	60
Боли в животе	0	0	30
Боли в костях	40	6	80
Проблемы при вставании из положения сидя	30	0	70

Анализ изменений качества жизни у пациентов после ПТЭ проведен с использованием метода «обобщенные уравнения оценки» (GEE) с учетом возможного влияния пола, возраста и исходного качества жизни на динамику показателей. На рис. 2 представлены скорректированный средний суммарный показатель по PHPQoL и скорректированные средние показатели качества жизни по шкалам SF-36 у больных ПГПТ до операции, через 3 мес и 12 мес после операции.

Выявлено статистически значимое увеличение суммарного показателя PHPQoL после операции (GEE, p<0,05). Также показано статистически значимое увеличение показателей по всем шкалам SF-36, кроме шкал Б и ОЗ, по сравнению с их исходными значениями до операции (GEE, p<0,05). Полученные результаты указывают на положительные изменения разных аспектов качества жизни пациентов после хирургического лечения — как специфических для ПГПТ, так и общих аспектов.

Суммарный показатель качества жизни по опроснику PHPQoL больных через 3 мес после операции увеличился на 12%, а через 12 мес — на 17% по сравнению с исходным значением (скорректированный средний показатель до операции 53,1 балла против 59,5 балла через 3 мес после операции и 62 балла через 12 мес после операции). При этом произошло улучшение как по физическому, так и психическому компонентам качества жизни. Через 3 мес и 12 мес показатель физического компонента качества жизни увеличился соответственно от 47,5±21,2 до 49,4±25,1 и 54,9±24,8 балла, а психического — от 47,0±19,1 до 51,5±23,4 и 57,6±24,8 балла.

На рис. 3 представлено распределение больных согласно суммарному показателю качества жизни по PHPQoL через 3 и 12 мес после ПТЭ в сравнении с его предоперационным значением. В связи с тем, что электронные формы опросников на динамических точках заполняли не все пациенты, в сравнительный анализ через 3 мес (а) и через 12 мес (б) включены разные пациенты.

**Figure:**
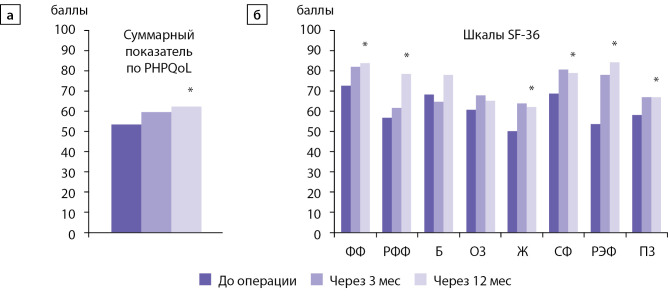
Рисунок 2. Скорректированный суммарный показатель опросника PHPQoL (а) и скорректированные средние показатели качества жизни по шкалам опросника SF-36 (б) у больных первичным гиперпаратиреозом до операции, через 3 и 12 мес после операции; *p<0,05.

**Figure:**
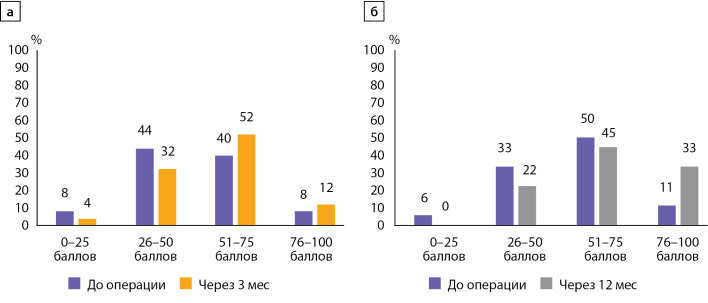
Рисунок 3. Распределение пациентов согласно величине суммарного показателя качества жизни по опроснику PHPQoL в группе больных первичным гиперпаратиреозом до операции и через 3 мес после операции (а), до операции и через 12 мес после операции (б).Примечание: анализ распределения пациентов через 3 и 12 мес после операции по сравнению с исходными показателями был проведен в разных группах больных в связи с тем, что не все пациенты заполняли электронные формы опросников в указанные сроки.

Как видно из рисунка, после операции уменьшилась доля больных с низкими баллами и увеличилась доля больных с высокими баллами качества жизни, хотя различия статистически не значимы (p>0,05). Отметим, что у большинства больных суммарный показатель качества жизни по PHPQoL через 3 и 12 мес после операции регистрировался в диапазоне 51–75 баллов (64 и 78% соответственно).

Клинически значимое улучшение качества жизни зарегистрировано у половины (48,5%) прооперированных больных, заполнивших опросник в динамике. При анализе групп с наличием и отсутствием клинически значимого улучшения качества жизни не установлены различия в распределении пациентов в группах согласно форме ПГПТ, уровню гиперкальциемии и выраженности актуальных симптомов до операции (χ2, p>0,05).

Установлена статистически значимая отрицательная корреляционная связь слабой силы между наличием клинически значимого улучшения качества жизни у пациентов после операции и исходным суммарным показателем по PHPQoL до операции (r=-0,376; p=0,031). Иными словами, чем меньше суммарный показатель до операции (чем хуже качество жизни), тем больше вероятность значимого для пациента улучшения качества жизни после ПТЭ, и наоборот. Связь между остальными исходными показателями, в том числе уровнем гиперкальциемии до операции и клинически значимым улучшением качества жизни, не обнаружена (p>0,05).

Отметим, что нами был также проведен анализ изменений качества жизни по данным опросников PHPQoL (табл. 3 и 4) и SF-36 (табл. 5 и 6) отдельно в группах с малосимптомной (n=8) и манифестной (n=17) формами ПГПТ. В соответствии с результатами анализа у пациентов с манифестной формой выявлена выраженная положительная динамика качества жизни после операции. Суммарный балл PHPQoL значительно увеличился через 3 мес после ПТЭ (средний эффект). Через 12 мес после операции имело место дальнейшее увеличение суммарного балла (большой эффект). Выраженную положительную динамику наблюдали через 3 мес после ПТЭ по всем шкалам SF-36, кроме шкалы боли, и через 12 мес — по всем шкалам SF-36. В группе с малосимптомной формой ПГПТ также установлена положительная динамика показателей через 3 и 12 мес после ПТЭ как по опроснику PHPQoL, так и по опроснику SF-36, однако данные изменения выражены в меньшей степени, чем в группе с манифестной формой заболевания. Для пациентов с малосимптомной формой ПГПТ положительные изменения качества жизни после операции отмечены в большей степени за счет улучшения по шкалам ролевого эмоционального функционирования, боли, физического функционирования и жизнеспособности; для пациентов с манифестной формой — по шкалам ролевого физического и ролевого эмоционального функционирования, боли и физического функционирования. Таким образом, как при манифестной, так и при малосимптомной формах ПГПТ, наблюдали улучшение качества жизни через 3 мес после ПТЭ. Положительная динамика сохранялась в обеих группах через год после операции.

**Table:** Таблица 3. Характеристика изменений качества жизни у больных с манифестной формой первичного гиперпаратиреоза через 3 и 12 мес после операции в сравнении с исходными показателями до операции по опроснику PHPQoL

Показатель	Изменения показателей через 3 мес в сравнении с исходными значениями*	Изменения показателей через 12 мес в сравнении с исходными значениями*
До операции	Через3 мес	∆ (величина эффекта, ES)	До операции	Через 12 мес	∆ (величина эффекта, ES)
Суммарный показатель	45,5	55,8	10,3 (0,7)	45,5	64,3	18,8 (0,9)
ФК	46,5	52,2	5,7 (0,4)	44,0	56,1	12,1 (0,6)
ПК	44,1	53,5	9,4 (0,6)	47,7	56,8	9,1 (0,6)

**Table:** Таблица 4. Характеристика изменений качества жизни у больных с малосимптомной формой первичного гиперпаратиреоза через 3 и 12 мес после операции в сравнении с исходными показателями до операции по опроснику PHPQoL

Показатель	Изменения показателей через 3 мес в сравнении с исходными значениями*	Изменения показателей через 12 мес в сравнении с исходными значениями*
До операции	Через3 мес	∆ (величина эффекта, ES)	До операции	Через 12 мес	∆ (величина эффекта, ES)
Суммарный показатель	53,9	59,1	5,2 (0,3)	53,9	60,0	6,1 (0,3)
ФК	54,2	58,4	4,2 (0,2)	55,4	60,1	4,7 (0,2)
ПК	53,6	60,1	6,5 (0,7)	52,4	64,7	12,3 (0,7)

**Table:** Таблица 5. Характеристика изменений качества жизни у больных с манифестной формой первичного гиперпаратиреоза через 3 и 12 мес после операции в сравнении с исходными показателями до операции по опроснику SF-36

Показатель	Изменения показателей через 3 мес в сравнении с исходными значениями*	Изменения показателей через 12 мес в сравнении с исходными значениями*
До операции	Через3 мес	∆ (величина эффекта, ES)	До операции	Через 12 мес	∆ (величина эффекта, ES)
ФФ	68,6	80,0	11,4 (0,6)	59,0	82,5	23,5 (0,9)
РФФ	36,8	57,4	20,6 (0,4)	30,6	72,8	42,2 (1,0)
Б	63,3	61,2	-2,1** (-)	61,4	81,7	20,3 (0,5)
ОЗ	57,8	65,3	7,5 (0,5)	54,4	71,7	17,3 (1,2)
Ж	42,2	61,2	19,0 (0,8)	45,8	64,1	18,3 (0,9)
СФ	64,7	80,9	16,2 (0,7)	66,1	79,6	13,5 (0,3)
РЭФ	41,7	77,1	35,4 (0,6)	40,0	75,8	35,8 (0,8)
ПЗ	55,8	67,0	11,2 (0,5)	52,7	65,5	12,8 (0,5)

**Table:** Таблица 6. Характеристика изменений качества жизни у больных с малосимптомной формой первичного гиперпаратиреоза через 3 и 12 мес после операции в сравнении с исходными показателями до операции по опроснику SF-36

Показатель	Изменения показателей через 3 мес в сравнении с исходными значениями*	Изменения показателей через 12 мес в сравнении с исходными значениями*
До операции	Через3 мес	∆ (величина эффекта, ES)	До операции	Через 12 мес	∆ (величина эффекта, ES)
ФФ	74,4	81,4	7,0 (0,4)	76,8	84,3	7,5 (0,4)
РФФ	64,3	64,3	0 (-)	62,5	64,3	1,8 (-)
Б	66,3	77,2	10,9 (0,3)	53,7	70,2	16,5 (1,9)
ОЗ	63,4	68,0	4,6 (0,2)	63,3	62,8	-0,5** (-)
Ж	55,0	62,1	7,1 (0,3)	52,5	57,9	5,4 (0,2)
СФ	71,4	78,5	7,1 (0,2)	70,8	73,2	2,4 (0,1)
РЭФ	50,0	61,9	11,9 (0,3)	52,8	71,5	18,7 (0,4)
ПЗ	64,3	66,9	2,6 (0,2)	59,7	57,2	-2,5** (0,1)

Дополнительно проведен анализ изменения выраженности актуальных симптомов у больных ПГПТ — слабости, усталости, боли в суставах, боли в костях, забывчивости и перемены настроения. На рис. 4 представлены скорректированные средние показатели их выраженности до, через 3 и 12 мес после операции (метод «обобщенные уравнения оценки», GEE).

Выявлено статистически значимое уменьшение усталости, слабости, забывчивости и перемен в настроении в течение 12 мес после операции (GEE, p<0,05). Через 12 мес после ПТЭ усталость составила 39 против 60,5 балла до операции, слабость — 32,7 балла против 52,2 балла, забывчивость — 31,4 против 43,3 балла, перемены в настроении — 22,5 против 33,8 балла (рис. 4).

**Figure:**
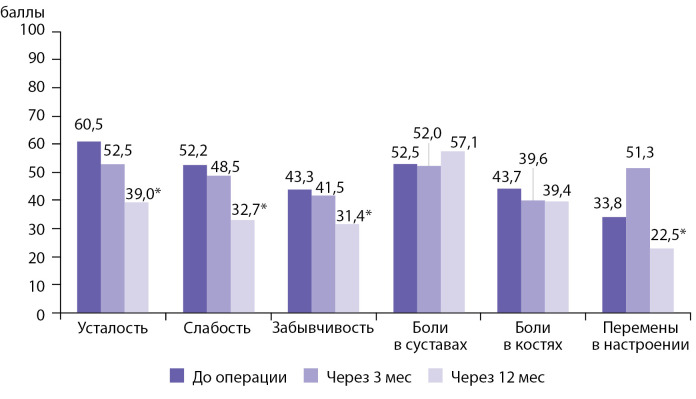
Рисунок 4. Скорректированные средние показатели выраженности актуальных симптомов у больных первичным гиперпаратиреозом до операции, через 3 мес и 12 мес после операции; *p<0,05.

Также отдельно провели анализ динамики симптомов в группах с манифестной и малосимптомной формами ПГПТ (табл. 7 и 8).

**Table:** Таблица 7. Характеристика изменений симптомов у больных с манифестной формой первичного гиперпаратиреоза через 3 и 12 мес после операции

Показатель	Изменения выраженности через 3 мес в сравнении с исходными значениями	Изменения выраженности через 12 мес в сравнении с исходными значениями
До операции	Через3 мес	∆ (величина эффекта, ES)	До операции	Через 12 мес	∆ (величина эффекта, ES)
Усталость	67,2	53,4	-13,8*(-0,36)	65,0	38,0	-27,0* (-0,61)
Слабость	64,4	54,6	-9,8* (-0,24)	58,0	33,3	-24,7* (-0,69)
Забывчивость	59,7	50,0	-9,7* (-0,27)	56,0	26,5	-29,5* (-0,52)
Боли в суставах	51,1	58,8	7,7 (0,17)	60,0	64,0	4,0 (0,08)
Боли в костях	47,8	46,7	-1,1* (-0,02)	54,0	52,0	-2,0* (-0,04)
Перемены в настроении	34,7	39,1	4,4 (0,18)	43,3	31,0	-12,3* (-0,43)

**Table:** Таблица 8. Характеристика изменений симптомов у больных с малосимптомной формой первичного гиперпаратиреоза через 3 и 12 мес после операции

Показатель	Изменения выраженности через 3 мес в сравнении с исходными значениями	Изменения выраженности через 12 мес в сравнении с исходными значениями
До операции	Через3 мес	∆ (величина эффекта, ES)	До операции	Через 12 мес	∆ (величина эффекта, ES)
Усталость	54,4	51,7	-2,7* (-0,2)	59,3	43,6	-15,7* (-0,3)
Слабость	40,0	42,9	2,9 (0,08)	48,6	29,3	-19,3* (-0,28)
Забывчивость	41,9	38,6	-3,3* (-0,08)	49,3	43,6	-5,7* (-0,08)
Боли в суставах	44,4	34,3	-10,1* (-0,25)	34,2	46,5	12,3 (0,24)
Боли в костях	29,6;	20,7	-8,9* (-0,46)	32,4	25,7	-6,7* (-0,23)
Перемены в настроении	28,1	48,5	20,4 (0,89)	36,4	25,0	-11,4* (-0,3)

В соответствии с данными табл. 7 и 8, и в группе с манифестной формой болезни, и в группе с малосимптомной формой ПГПТ наблюдали уменьшение усталости, слабости, забывчивости. При этом следует отметить, что у пациентов с малосимптомной формой ПГПТ уменьшение данных симптомов выражено в меньшей степени, чем у пациентов с манифестной формой. Примечательно, что у пациентов с малосимптомной формой выявлено также уменьшение болей в костях через 12 мес после операции.

Направления дальнейших исследований

Актуальные направления для дальнейшей исследовательской работы — изучение особенностей динамики качества жизни у больных ПГПТ с манифестной и малосимптомной формами заболевания в отдаленные сроки после оперативного лечения, выявление факторов, связанных с пациентом, которые влияют на исходы операции у больных ПГПТ, а также определение связи эффекта ПТЭ с клиническими показателями и качеством жизни пациентов до операции.

## ОБСУЖДЕНИЕ

В рамках проспективного наблюдательного исследования на ограниченной популяции пациентов с ПГПТ проведен анализ изменения показателей качества жизни у больных на протяжении 12 мес после успешной ПТЭ по сравнению с исходными значениями до операции. Выборка, состоящая из 72 больных ПГПТ, включала пациентов как с манифестной, так и с малосимптомной формами ПГПТ, с разной длительностью заболевания и разным уровнем лабораторных показателей, характерных для ПГПТ, и соответствовала условиям реальной клинической практики. Данные, проанализированные до операции, демонстрируют общее снижение качества жизни пациентов с ПГПТ по сравнению с условно здоровыми респондентами и отражают наличие специфических проблем у больных данного профиля. У половины пациентов выявлены такие существенно выраженные симптомы, как слабость, усталость, когнитивные нарушения, перемены в настроении, а также суставные и костные боли; продемонстрирована связь между испытываемыми симптомами и степенью нарушения качества жизни у больных ПГПТ до операции. Полученные результаты сопоставимы с опубликованными данными зарубежных исследований [9–12][14]. Так, в наблюдательном исследовании Webb S.M. и соавт. в группе 182 пациентов с ПГПТ (средний возраст 60,3±11,7 года, 78% — женщины) было показано, что чаще всего пациенты испытывали такие симптомы, как слабость (48,4%), боли в костях (47,8%) и боли в мышцах или мышечную слабость (42,9%); количество симптомов коррелировало со степенью нарушения качества жизни по PHPQoL (r=0,55; p<0,001), при этом у пациентов с манифестной формой заболевания качество жизни было хуже, чем у пациентов с малосимптомной формой (p<0,001) [14].

После проведенного хирургического вмешательства наблюдались положительные изменения качества жизни уже через 3 мес после операции, которые сохранялись в течение 12 мес. Улучшение качества жизни у больных ПГПТ после хирургического лечения показано авторами в разных работах, причем как в ранние сроки после операции, так и при длительном наблюдении [10][11][12][14]. В работе Ryhanen E.M. и соавт. [10] установлено, что в группе из 124 больных ПГПТ (средний возраст 65,0±10,2 года, 82% — женщины) суммарный балл качества жизни по общему опроснику 15D до операции был значительно ниже в сравнении с таковым у респондентов из общей популяции (0,813 против 0,904, p<0,001). Однако через 6 мес после операции этот показатель существенно увеличился (0,865), в течение последующих 6 мес происходило его дальнейшее значимое увеличение до 0,878 через 12 мес после операции (p<0,001). Полученные в нашем исследовании данные с применением другого общего опросника — SF-36 также продемонстрировали, что у больных ПГПТ до операции показатели качества жизни существенно снижены в сравнении с таковыми у условно здоровых лиц, а через 3 и 12 мес после ПТЭ происходит их выраженная положительная динамика. В исследовании Somuncu E. и Kara Y. [12] при использовании специального опросника PHPQoL в группе из 41 больного ПГПТ (средний возраст 60,4 года, 80,4% — женщины) показано улучшение качества жизни после операции: суммарный показатель по опроснику до операции составил 48±22 балла, через 12 мес — 62±24 балла (p<0,05). Согласно данным нашего исследования, суммарный показатель по опроснику PHPQoL также существенно увеличился после ПТЭ: 53,1 балла до операции и 62 балла через 12 мес после операции.

Особенность нашего исследования связана с тем, что данная работа выполнена в соответствии с современной методологией оценки качества жизни при использовании стандартизированных опросников — как общего, так и специальных опросников для больных ПГПТ. Применение общего и специального опросников позволяет выполнить полноформатную оценку как основных составляющих качества жизни, так и его специфических аспектов при данном заболевании в процессе лечения. Дополнительным преимуществом данного исследования является применение современных статистических подходов, позволяющих учитывать влияние разных факторов, в том числе пола, возраста, исходного качества жизни, на изменение показателей во времени с помощью построения обобщенных оценочных моделей (GEE). Такой подход наиболее корректен и соответствует современным рекомендациям по проведению анализа данных качества жизни, меняющихся во времени и связанных с различными факторами.

В рамках исследования показано улучшение как по физическому, так и психическому компонентам качества жизни у больных ПГПТ после операции. Положительные изменения качества жизни установлены как при манифестной, так и при малосимптомной форме заболевания. У большинства больных суммарный показатель качества жизни по PHPQoL через 3 мес и через 12 мес после операции регистрировался в диапазоне высоких значений — от 51 до 75 баллов. Значимое для пациента улучшение качества жизни показано у 48,5% прооперированных больных. В течение 12 мес после операции отмечено существенное уменьшение таких актуальных для ПГПТ симптомов, как слабость, усталость, забывчивость и перемены в настроении. В целом полученные результаты демонстрируют эффект хирургического лечения с точки зрения пациента и сопоставимы с опубликованными данными [7–13]. Отметим также, что динамика качества жизни не была связана с таким важным показателем, как исходный уровень гиперкальциемии. Похожие результаты, согласно которым улучшение качества жизни после ПТЭ наблюдалось у пациентов с разным уровнем гиперкальциемии до операции, были продемонстрированы в других работах [11][12][14]. Необходимы дальнейшие исследования для углубленного изучения связи эффекта ПТЭ, в том числе в отдаленные сроки после операции, с клиническими показателями, в первую очередь уровнем гиперкальциемии.

Наше исследование имеет несколько ограничений. К ним следует отнести относительно небольшое количество наблюдений и включение в исследование пациентов из одного центра. Также ограничением исследования является проведение анализа данных на динамических точках только у тех пациентов, у которых была возможность дистанционного заполнения опросников. Дополнительно отметим некоторое смещение нашей выборки относительно возраста и пола (средний возраст меньше, чем в других исследованиях, а также очень мала доля пациентов мужского пола), что требует осторожности при сравнении полученных результатов с данными других исследований. Кроме того, длительность наблюдения ограничена сроком 12 мес, что также необходимо учитывать при проведении дальнейших исследований с целью уточнения изменений качества жизни и симптомов в отдаленные сроки после операции. По мнению специалистов-эндокринологов, в настоящее время перспективной и важной остается задача мониторинга состояния больных ПГПТ в отдаленный период после лечения с использованием современных стандартизированных опросников, позволяющих получить полноформатную информацию о нарушении разных аспектов качества жизни до и после проведенного лечения [11][17][19].

В целом полученные нами данные подтверждают важность учета информации о качестве жизни пациента для определения соотношения рисков и пользы при выборе хирургической тактики лечения наряду с оценкой клинических показателей у больных ПГПТ как при манифестном, так и при малосимптомном течении заболевания. Информация о качестве жизни, полученная напрямую от пациента на основании стандартизированных опросников, может существенно дополнить клиническую информацию о больном, которой располагает врач-эндокринолог, способствовать реализации пациент-ориентированного подхода при ведении больных ПГПТ хирургического профиля и в целом совершенствованию качества медицинской помощи данной категории пациентов. Также следует отметить, что мониторинг качества жизни и симптомов на основе электронных опросников может предоставить дополнительное преимущество при дистанционном наблюдении за состоянием прооперированных пациентов в период пандемии COVID-19.

## ЗАКЛЮЧЕНИЕ

Качество жизни больных ПГПТ до операции отличается выраженным нарушением. У 43% пациентов имелось низкое или очень низкое качество жизни (менее 50 баллов согласно специальному опроснику для больных ПГПТ — PHPQoL). Нарушение качества жизни по опроснику SF-36 до операции в большей степени наблюдали со стороны ролевого функционирования, физического и социального функционирования, а также жизнеспособности, что обусловлено влиянием заболевания и связано с такими специфическими проблемами у более половины пациентов, как слабость, усталость, боли в костях и суставах, изменения в когнитивной и психологической сферах.

Успешное хирургическое лечение с нормализацией уровня паратиреоидного обмена способствует улучшению качества жизни больных ПГПТ, независимо от формы заболевания. Согласно данным нашего исследования, средний суммарный показатель по опроснику PHPQoL существенно увеличился после ПТЭ: 53,1 балла до операции и 62 балла через 12 мес после операции. Положительная динамика качества жизни сохранялась через год после операции как у больных с манифестной, так и малосимптомной формами болезни. Для пациентов с малосимптомной формой ПГПТ положительные изменения после операции были отмечены в большей степени за счет улучшения физического функционирования, ролевого эмоционального функционирования и жизнеспособности, а также снижения выраженности боли. У пациентов с манифестной формой положительные изменения в большей степени касались физического функционирования, ролевого функционирования (как физического, так и эмоционального) и снижения уровня боли. Через 3 и 12 мес после ПТЭ установлено существенное уменьшение актуальных симптомов у пациентов данного профиля, таких как усталость, слабость, забывчивость и перемены настроения; изменения выражены в большей степени у больных с манифестной формой ПГПТ, однако при малосимптомной форме болезни также имелась тенденция к уменьшению данных симптомов.

Ухудшение качества жизни у пациентов с бессимптомным (малосимптомным) ПГПТ является серьезным аргументом в пользу определения показаний к хирургическому лечению этой формы заболевания.

Использование информации, полученной на основании опросников SF-36, PHPQoL и PAS до и после хирургического вмешательства, позволяет мониторировать изменения качества жизни больных ПГПТ и оценивать динамику физического и психологического функционирования, отслеживать изменение специфических аспектов качества жизни, нарушенных вследствие заболевания, а также осуществлять контроль актуальных симптомов у больных ПГПТ после проведенного лечения.

Применение стандартизированных опросников оценки качества жизни и симптомов позволяет персонифицировать клинический подход при ведении больных ПГПТ, открывает возможность получать ценную информацию о проблемах, связанных с данным заболеванием и лечением, напрямую от пациента и использовать ее как на этапе принятия решения при выборе хирургической тактики, так и в составе комплексной оценки эффективности терапии при определении степени восстановления разных аспектов функционирования у пациентов после операции.

## ДОПОЛНИТЕЛЬНАЯ ИНФОРМАЦИЯ

Источники финансирования. Работа выполнена по инициативе авторов без привлечения финансирования.

Конфликт интересов. Авторы заявляют об отсутствии конфликта интересов.

Участие авторов. Русаков В.Ф., Черников Р.А., Ионова Т.И., Ефремов С.М. — идея и дизайн исследования; Карелина Ю.В. — предоставление материалов исследования; Гладкова И.Н. — сбор данных, формирование деперсонифицированной выборки пациентов; Никитина Т.П. — формирование и ведение базы данных; Никитина Т.П., Ионова Т.И. — анализ и интерпретация данных, написание текста рукописи; Русаков В.Ф., Черников Р.А., Ефремов С.М., Ионова Т.И., Никитина Т.П. — финальный анализ и редактирование текста рукописи. Все авторы внесли существенный вклад в проведение исследования и подготовку статьи, прочли и одобрили финальную версию перед публикацией.

## ПРИЛОЖЕНИЕ 1

Опросник оценки качества жизни при первичном гиперпаратиреозе (PHPQoL)

Вопросы0 — всегда1 — очень часто2 —время от времени3 — очень редко4 — никогда1.Я испытывал(а) сонливость после того, как просыпался(ась) утром, и мне было трудно начинать день     2.Я ощущал(а) слабость     3.Я замечал(а), что мне тяжело долго ходить     4.Я замечал(а), что задыхаюсь, если мне приходится идти быстро     5.Я испытывал(а) боль в спине     6.У меня болели кости и суставы     7.Я замечал(а), что мне тяжело выполнять свои ежедневные дела     8.Я ограничивал(а) свой досуг     9.Я ограничивал(а) свои домашние обязанности     10.Я чувствовал(а) раздражительность     11.Я бывал(а) в плохом настроении/депрессии     12.Из-за болезни я страдал(а) бессонницей     13.Я просыпался(ась) ночью     14.Я замечал(а), что мне бывает трудно сконцентрироваться     15.Я переживал(а) не только из-за болезни, но и из-за ее возможных осложнений     16.Я замечал(а), что мне бывает труднее сконцентрироваться на работе, чем раньше     

 
